# Coping with Salt Water Habitats: Metabolic and Oxidative Responses to Salt Intake in the Rufous-Collared Sparrow

**DOI:** 10.3389/fphys.2017.00654

**Published:** 2017-09-01

**Authors:** Pablo Sabat, Cristóbal Narváez, Isaac Peña-Villalobos, Carolina Contreras, Karin Maldonado, Juan C. Sanchez-Hernandez, Seth D. Newsome, Roberto Nespolo, Francisco Bozinovic

**Affiliations:** ^1^Departamento de Ciencias Ecológicas, Facultad de Ciencias, Universidad de Chile Santiago, Chile; ^2^Center of Applied Ecology and Sustainability, Pontificia Universidad Católica de Chile Santiago, Chile; ^3^Laboratory of Ecotoxicology, Faculty of Environmental Sciences and Biochemistry, University of Castilla-La Mancha Toledo, Spain; ^4^Department of Biology, University of New Mexico Albuquerque, NM, United States; ^5^Instituto de Ciencias Ambientales y Evolutivas, Facultad de Ciencias, Universidad Austral de Chile Valdivia, Chile; ^6^Departamento de Ecología, Facultad de Ciencias Biológicas, Pontificia Universidad Católica de Chile Santiago, Chile

**Keywords:** basal metabolic rate, birds, maximum metabolic rate, metabolism enzymes, oxidative stress, salt intake

## Abstract

Many physiological adjustments occur in response to salt intake in several marine taxa, which manifest at different scales from changes in the concentration of individual molecules to physical traits of whole organisms. Little is known about the influence of salinity on the distribution, physiological performance, and ecology of passerines; specifically, the impact of drinking water salinity on the oxidative status of birds has been largely ignored. In this study, we evaluated whether experimental variations in the salt intake of a widely-distributed passerine (*Zontotrichia capensis*) could generate differences in basal (BMR) and maximum metabolic rates (M_sum_), as well as affect metabolic enzyme activity and oxidative status. We measured rates of energy expenditure of birds after 30-d acclimation to drink salt (SW) or tap (fresh) water (TW) and assessed changes in the activity of mitochondrial enzymes (cytochrome c oxidase and citrate synthase) in skeletal muscle, heart, and kidney. Finally, we evaluated the oxidative status of bird tissues by means of total antioxidant capacity (TAC) and superoxide dismutase activities and lipid oxidative damage (Malondialdehyde, MDA). The results revealed a significant increase in BMR but not M_sum_, which resulted in a reduction in factorial aerobic scope in SW- vs. TW-acclimated birds. These changes were paralleled with increased kidney and intestine masses and catabolic activities in tissues, especially in pectoralis muscle. We also found that TAC and MDA concentrations were ~120 and ~400% higher, respectively in the liver of animals acclimated to the SW- vs. TW-treatment. Our study is the first to document changes in the oxidative status in birds that persistently drink saltwater, and shows that they undergo several physiological adjustments that range that range in scale from biochemical capacities (e.g., TAC and MDA) to whole organism traits (e.g., metabolic rates). We propose that the physiological changes observed in *Z. capensis* acclimated to saltwater could be common phenomena in birds and likely explain selection of prey containing little salt and habitats associated with low salinity.

## Introduction

The consumption of salt and its concomitant physiological cost represent a significant challenge to birds and may limit species range as well as colonization of new habitats (Nehls, 1996; Gutiérrez et al., 2011). These costs are due to the exacerbated functioning of the machinery required for metabolizing, processing, and excreting excess salts, and are usually characterized by an increase in the costs of organismal maintenance. In seabirds, these costs are offset by the osmotic work achieved by the salt gland (Nehls, 1996). For instance, Gutiérrez et al. (2012) found that basal metabolic rate (BMR) was significantly lower in inland shorebirds that had access to freshwater in comparison to their coastal counterparts. Few studies have empirically evaluated the direct energetic outcomes from salt-rich diets and saltwater in birds. Recently, Gutiérrez et al. (2011) showed experimentally that the maintenance of active osmoregulatory machinery by the Dunlin (*Calidris alpina*) is energetically expensive and was associated with an increase in metabolic rate by ~40%. Likewise, Peña-Villalobos et al. (2013) experimentally found a 30% increase in BMR in a small passerine, the rufous collared sparrow (*Zonotrichia capensis*), that drank saltwater for 30 days in comparison to control animals that drank fresh (tap) water.

Osmoregulatory work is the energy cost in a thermodynamic sense (Borsook and Winegarden, 1931) of using specialized organs (e.g., salt gland, kidneys) to concentrate and excrete salts. In birds, theoretical estimates of added osmoregulatory costs range from 1% in ducks (Peaker and Linzell, 1975) to 7% for sparrows that consumed saltwater (200 mM NaCl, Peña-Villalobos et al., 2013). These estimates are lower than the actual 30–40% increases in BMR observed in saltwater-acclimated animals (Gutiérrez et al., 2011; Peña-Villalobos et al., 2013). Thus, thermodynamically-derived estimates of osmotic work tend to underestimate the overall cost of salt excretion in birds, especially in species that lack functional salt glands (Peña-Villalobos et al., 2013). In contrast to marine adapted species, intake of concentrated fluids in terrestrial (passerine) birds is generally coupled with high urine flow (McNabb et al., 1972), which is a costly strategy for birds that live in dry environments (McWhorter et al., 2004). Therefore, hyperosmotic urine excretion in *Z. capensis* has been associated with BMR as well as increased kidney and heart mass (Peña-Villalobos et al., 2013). Accordingly, it is also likely that BMR would increase in saltwater-acclimated birds because kidney and other internal organs increase tissue-specific rates of energy expenditure via enhanced activity of mitochondrial enzymes.

Both osmoregulation and thermoregulation are closely associated physiological processes that make use of a common energetic budget in birds (e.g., Gutiérrez et al., 2012). The investment of energy toward activity, growth, and/or reproduction is only possible when energy intake exceeds maintenance requirements (Careau et al., 2014). Furthermore, the allocation model predicts a negative association between energy-demanding activities and BMR because animals should allocate a fixed amount of energy between those competing processes, which is especially acute when food intake becomes limited (Speakman, 1997; Careau et al., 2008). Because the excretion of excess salts is usually characterized by an increase in the cost of maintenance, osmoregulation could affect the amount of energy allocated to activity, which would have obvious consequences for fitness. Such energetic costs could also have a significant impact on thermoregulatory performance (Verboven and Piersma, 1995). Because maximum metabolic rate (M_sum_) and aerobic scope (the ratio and difference between M_sum_ and BMR, see methods) have been related to thermogenesis (Guderley and Pörtner, 2010; Bozinovic et al., 2011; Swanson and Bozinovic, 2011), an increase in osmoregulatory cost may have a significant impact on M_sum_ and aerobic scope with concomitant negative consequences on animals' thermogenesis and aerobic capabilities. At present, there are no empirical data to understand how these physiological traits interact.

Another potential cost of salt intake is the increased production of reactive oxygen species (ROS). Oxidative metabolism has been associated with ROS production, which leads to cumulative damage of molecules, such as DNA, proteins, and lipids that can result in increased disease risk and death (Dowling and Simmons, 2009; Monaghan et al., 2009; Costantini, 2010; van de Crommenacker et al., 2010; Selman et al., 2012). While it has been shown that increased osmoregulatory function is positively associated with stress in invertebrates and fish (Martínez-Álvarez et al., 2002; Tremblay and Abele, 2016; Velez et al., 2016; Rivera-Ingraham and Lignot, 2017), we do not currently understand how salt intake and changes in osmotic conditions may increase the ROS production and cause oxidative damage in birds. In addition to changes in metabolic rates and tissue biochemical activities, a common response to high osmotic loads is the increase in urine concentration mediated by a complex interplay of hormones (Braun and Dantzler, 1984; McCormick and Bradshaw, 2006). For example, plasma corticosterone and aldosterone concentrations increase following salt ingestion to maintain water and mineral balance (Phillips and Ensor, 1972; Harvey et al., 1984). Accordingly, marine bird species have higher baseline corticosterone concentrations than terrestrial birds (Brischoux et al., 2015). It has also been shown that corticosterone and other glucocorticoids disturb cellular oxidative homeostasis (Costantini et al., 2011; Queisser et al., 2011; Brand et al., 2014; Spiers et al., 2014). Specifically, chronic corticosterone administration to *Gallus gallus*, induced the formation of ROS and increased lipid peroxidation (Lin et al., 2004). Thus, it is likely that the ingestion of saltwater and associated osmotic stress could cause changes in the oxidative status in birds.

Here, we experimentally evaluated to what extent the differences in energy demands imposed by osmoregulatory work affects thermoregulatory function and oxidative status in a widely-distributed passerine (*Zonotrichia capensis*). Using an acclimation experiment, we assessed whether the animals exhibited metabolic and biochemical responses to salt intake by directly measuring mass-specific metabolic capabilities of the kidney and other metabolically-active organs, the energy allocated to thermoregulation (M_sum_ and aerobic scope), and biomarkers of oxidative stress to assess this species endurance to salt load. We predicted that animals acclimated to a higher salt load should (1) increase the activity of selected metabolism enzymes, (2) decrease thermoregulatory capabilities, and (3) exhibit higher levels of oxidative damage and/or elevated biochemical antioxidant mechanisms, compared with those acclimated to fresh water. By characterizing the physiological effects of consumption of saltwater via a laboratory experiment that measured a suite of biochemical and whole organism physiological traits, our results provide insights into the proximate factors responsible for the variability in the use of coastal habitats by terrestrial birds.

## Materials and methods

We used rufous-collared sparrows (*Z. capensis*), which are ubiquitous in Chile and occurs in a range of habitats across substantial altitudinal gradient from coastal to high elevation (e.g., 4,000 m.a.s.l.) alpine areas (Goodall et al., 1946). Previous experiments have shown that this species can tolerate drinking saline solutions of 200 mM NaCl without significant changes in its body weight, however, increases in BMR were observed when birds were exposed to such high salt loads (Peña-Villalobos et al., 2013). Birds (*n* = 18) were captured with mist nets in central Chile (33° 30′S, 70° 54′W) in the austral autumn (April and May). Following capture, birds were transported to the University of Chile using boxes to avoid stress during transport. In the laboratory, the birds were maintained in individual cages of 50 × 50 × 50 cm at 25 ± 2°C, and a L:D cycle of 12:12 h. During a 21-d habituation period to laboratory conditions, the sparrows consumed mealworms (*Tenebrio molitor*), birdseed and water, which were available *ad libitum*. Water was offered in graduated inverted plastic tubes of 100 mL. Birds then were divided into two experimental groups: nine birds received tap water (TW-acclimated group) and the other nine individuals received saltwater (SW-acclimated group) that contained 200 mM NaCl for 30 days. Both treatment groups were fed with mealworms and seeds *ad libitum*. After the acclimation period, non-anesthetized animals were gently contained with one hand, while a second person performed the procedure to obtain urine and blood. Blood samples (50–100 μL) were collected in the morning (09:00–11:00 h) from the humeral vein using heparinized tubes and samples were centrifuged at 9000 × g for 5 min. Plasma was separated from red blood cells and frozen at −80°C until analysis. Ureteral urine was obtained by inserting a small closed-ended cannula into the birds' cloaca. Urine samples from each bird were centrifuged and the supernatant was frozen (−80°C) for osmometry analysis (Wescor 5130B). At the end of the experiment, daily fluid intake rates were measured using graduated inverted plastic tubes of 100 mL and corrected for evaporation by using control tubes located outside the cage.

Metabolic rates were estimated through oxygen consumption rate (VO_2_) using a FoxBox respirometer (Sable Systems, Las Vegas, NV). For BMR, animals were fasted for 4 h, weighed and then placed in a dark metabolic chamber (2 L) located in a controlled temperature cabinet (Sable Systems, Henderson, Nevada) at a constant ambient temperature (*Ta* = 30 ± 0.5°C). We passed both incurrent and excurrent gas streams through columns of Drierite and Baralyme to remove H_2_O and CO_2_, maintaining flow rates at 500 mL min^−1^ with the mass-flow controller included in the FoxBox. Output from the oxygen analyzer (%) was digitalized using a Universal Interface II (Sable Systems) and recorded on a computer using EXPEDATA data acquisition software (Sable Systems). Our sampling interval was 5 s. All measurements were made during the resting phase between 18:00 and 07:00 h. Because water vapor and CO_2_ were removed before entering the O_2_ analyzer, and the flow rates are measured downstream from the metabolic chamber, oxygen consumption was calculated according to equation (11.1) in Lighton (2008) as:

(1) $$VO_{2}=\;\frac{FR\;\times \;60\;\times \;(Fi\;O_{2}-\;Fe\;O_{2})}{1-Fi\;O_{2}}$$

where FR is the flow rate in ml min^−1^, and FiO_2_ and FeO_2_ are the fractional concentrations of inflow and outflow O_2_ in the metabolic chamber, respectively. Maximum metabolism (*M*_sum_) was determined in a He-O_2_ (80–20%, INDURA, Chile) atmosphere according to Rosenmann and Morrison (1974) at −5 ± 2°C, following a similar protocol used for BMR (Narváez et al., 2016). Measurements were stopped when a decrease of VO_2_ consumption was evident after a visual inspection of the data. To verify that animals were hypothermic, body temperature (Tb) was checked with an intra rectal thermocouple (± 0.1°C) after each measurement. Only measurements with hypothermic birds were considered (i.e., *Tb* < 35°C). We recorded the time to hypothermia, as an indicator of thermogenic endurance under cold stress (Swanson, 2001). Oxygen consumption during trials was calculated as instantaneous VO_2_ from readings taking every 5 s, and we considered M_sum_ as the highest 5-min average oxygen consumption over the test period (Swanson and Bozinovic, 2011).

To test the biochemical adjustments of internal organs to salinity, we measured mitochondrial enzyme activity in all individuals after the acclimation period. Birds were sacrificed by CO_2_ exposure, weighed and dissected to remove the organs. Liver, kidney and heart samples were first homogenized and protein concentrations were determined using the method by Bradford method (Bradford, 1976), with bovine serum albumin as the standard. We measured the activity of two mitochondrial enzymes: (a) cytochrome c oxidase (COX; E.C. 1.9.3.1), an enzyme involved in the last reaction of the mitochondrial respiratory chain, which is indicative of the energy capacity of the mitochondrial system, and (b) citrate synthase (CS; E.C. 4.1.3.7), an enzyme participating in Krebs cycle function. An increase in the activity of these enzymes likely reflects changes in both the functional properties and the density of mitochondria (Guderley, 1998). COX activity was measured using a spectrophotometric method slightly modified from that by Moyes et al. (1997). Briefly, enzyme activity was measured in a reaction mixture containing 10 mM Tris-HCl (pH 7), 120 mM KCl, 250 mM sucrose, and cytochrome c reduced with dithiothreitol in a final volume of 0.2 ml. Enzyme activity was calculated using an extinction coefficient of 21.84 mM^−1^ cm^−1^ at 550 nm for cytochrome-c. CS activity was measured according to Sidell et al. (1987) with slight modifications. The enzyme assay medium contained 10 mM Tris-HCl (pH 8.0), 10 mM 5,5′dithiobis- (2•nitrobenzoic acid), 30 mM acetyl Coenzyme A (acetyl CoA) and 10 mM oxaloacetic acid (OAA; omitted in the controls) in a final volume of 0.2 mL. Enzyme activity was calculated using an extinction coefficient of 13.6 mM^−1^ cm^−1^ at 412 nm. For both enzymes, the extinction coefficient was monitored with a Thermo Scientific Multiscan monochromator-based UV/VIS spectrophotometer at 25°C. All enzyme activities are reported as specific activity per milligram of protein (μmol min^−1^ mg^−1^).

### Oxidative status

Oxidative status was assessed by the measurement of four biomarkers: (1) the enzyme superoxide dismutase (SOD, EC 1.15.1.1) as a biochemical antioxidant mechanism, (2) the concentration of the free radical nitric oxide (NO), (3) the total antioxidant capacity (TAC) that is a measurement of the presence of molecular antioxidants in tissues, and (4) lipid peroxidation as a measurement of oxidative damage.

### Superoxide dismutase

Tissues were suspended with phosphate-buffered saline (PBS), and then diluted (10x) and homogenized in 0.1 M Tris-HCl pH 7.4 buffer containing 0.5% Triton X-100, 5 mM β-mercaptohethanol (ME), 0.1 mg / mL phenylmethylsulfonyl fluoride (PMSF) and then centrifuged at 14,000 g for 5 min at 4°C. Blood was collected as described above. Superoxide dismutase activity was determined by the formazan method (Peskin and Winterbourn, 2000) using a commercially available kit (Biovision, Milpitas, CA; #K335). The kit uses a water-soluble tetrazolium salt (WST-1), which produces a water-soluble formazan with the reduction of the superoxide anion. This rate of reduction with the superoxide anion is linearly related to the activity of xanthine oxidase, and is inhibited by SOD. The inhibition activity of SOD was then measured by changes in extinction at 450 nm in the Multiskan at 25°C. The percentage of inhibition is then obtained from the SOD calibration curve to obtain the enzymatic units as a function of the percentage of inhibition.

### Nitric oxide

Tissues were diluted (10 x), homogenized with PBS and centrifuged at 12,000 rpm for 10 min at 4°C. After discarding the pellet, the homogenate was again centrifuged using 10kDa filters. Blood was collected with EDTA and centrifuged at 10,000 rpm for 10 min at 4°C. The plasma was again centrifuged and passed through a 10 kDa filter. It was then frozen immediately at −80°C for subsequent measurements. Nitric oxide (NO) concentration was measured according to Patton and Kryskalla (2011) using a commercially available kit (Biovision, Milpitas, CA; #K262) and monitored at 540 nm with the Thermo Scientific Multiskan.

### Total antioxidant capacity

A sample of each tissue was diluted 10x and homogenized with PBS, then centrifuged at 10,000 g for 10 min at 4°C. The supernatant was immediately stored at −80°C until assay were conducted. Blood was collected with heparin and centrifuged at 10,000 g for 10 min at 4°C. Finally, plasma was removed, and stored at −80°C for further analysis. We used the reducing antioxidant capacity method (Apak et al., 2004) to assess total antioxidant capacity (TAC). The assay kit produced by Cell Biolabs OxiSelect™, (San Diego, CA; # STA-360) is based on the production of a chromogenic reagent with a maximum absorbance at 490 nm.

### Lipid peroxidation

A sample of each tissue was washed with PBS containing heparin and diluted 10-fold with PBS containing 5% Butylated hidroxytoluene (BHT) to prevent oxidation. Tissues were homogenized and centrifuged at 10,000 g for 5 min at 4°C. The supernatant was removed and stored at −80°C until measurement. Blood was collected with heparin and then centrifuged at 10,000 g for 5 min at 4°C, then 5% BHT was added to prevent oxidation and stored at −80°C until measurement. Lipid peroxidation was assessed via measurement of thiobartbituric acid as described by Ohkawa et al. (1979). The assay kit test by Cell Biolabs OxiSelect™ (San Diego, CA; # STA-330) evaluates a 1:2 adduct formed between Malondialdehyde (MDA) and thiobarbituric acid (TBA); The MDA-TBA adduct can be determined by colorimetry at 532 nm.

### Statistical analysis

We tested the effect of acclimation condition on physiological (i.e., time to hypothermia, BMR and M_sum_) and morphological traits using MANCOVA with body mass as co-variable even though our experimental groups did not differ in mass (see Table 1). When an analysis revealed a non-significant effect of body mass, this term was dropped from the model. We calculated two measures of aerobic scope: factorial aerobic scope (FAS: M_sum_/BMR) and net aerobic scope (NAS: M_sum_-BMR), which are measures of aerobic capacity. Oxidative stress parameters and metabolic enzymes were evaluated using repeated-measures ANOVA with acclimation condition as single factor, and values of each response variable in each tissue as repeated measures within each individual. To compare the oxidative stress biomarkers between tissues, we standardized all data to mg of total protein for each tissue. Degrees of freedom of all statistical analyses may differ because in some cases the number of tissues analyzed differed among the biochemical parameters evaluated. To test for specific differences among means in physiological, morphological, and biochemical traits we used a *post hoc* Fisher test. Prior to each statistical analysis, data were examined for assumptions of normality and homogeneity of variance using Kolmogorov–Smirnov and Levene tests, respectively. For variables that were not normally distributed, we used logarithmic transformed data for statistical analysis.

**Table 1:** Body mass, organ mass, and osmolality of fluids (means ± *SD*) of *Zonotrichia capensis* acclimated to tap (fresh) and salt water (200 mM NaCl).

	**Salt water**	**Tap (fresh) water**
Body mass (g)	20.93 ± 1.08	20.66 ± 1.30
Pectoralis (g)	2.26 ± 0.29	2.21 ± 0.38
Liver mass (g)	0.64 ± 0.09	0.57 ± 0.08
Gizzard (g)	0.82 ± 0.16	0.74 ± 0.08
Heart mass (g)	0.28 ± 0.04	0.28 ± 0.02
Kidney mass (g)	0.27 ± 0.02^*^	0.21 ± 0.01
Intestine mass (g)	0.79 ± 0.41	0.57 ± 0.15
Large intestine mass (mg)	51.2 ± 7.13^*^	38.30 ± 7.4
Small intestine length (cm)	13.52 ± 1.66	13.17 ± 1.06
Large intestine length (cm)	1.09 ± 0.15	1.16 ± 0.22
Plasma Osmolality (mOsm/Kg)	325.7 ± 6.35	329.0 ± 12.3
Urine Osmolality (mOsm/Kg)	385.1 ± 19.7^*^	303.7 ± 26.72
Water intake (mL/h)	0.51 ± 0.3	0.41 ± 0.22
Time to Hypothermia (min)	52.8 ± 17.8	60.1 ± 12.8

*Asterisk denotes significant differences between treatments (see text for statistical output)*.

### Ethics statement

This study was carried out in accordance with the recommendations of the guide “Regulation of the use and care of experimental animals” of the Bioethics Committee, Comisión Nacional de Investigación Científica y Tecnológica (CONICYT). The protocol was approved by the Institutional Animal Care Committee of the University of Chile.

## Results

### Morphology

Birds maintained body mass during the period of habituation to laboratory conditions [Paired *t*-test: *t*_(17)_ = 0.05, *p* = 0.96]. In addition, body mass did not significantly differ from the capture weight after 1 month of acclimation either to salt or tap water conditions; *t*_(5)_ = 1.59, *p* = 0.17; *t*_(8)_ = 1.35, *p* = 0.20 for TW- and SW-acclimated birds, respectively.

MANCOVA analysis revealed a significant effect of treatment on all morphological variables [Wilks lambda = 0.14, *F*_(9, 7)_ = 4.80, *p* = 0.025]. *A posteriori* analyses revealed that SW-acclimated birds had larger intestine (*p* = 0.003) and kidney (*p* = 0.0004) mass than TW-acclimated birds, but there was no effect of drinking water treatment on liver (*p* = 0.12), small intestine (*p* = 0.25), gizzard (*p* = 0.30), pectoralis muscle (*p* = 0.77), or heart (*p* = 0.81) mass. The length of the small and large intestine did not differ between treatment (*p* = 0.64 and *p* = 0.41, respectively. At the end of acclimation period, water intake was similar between treatments [*F*_(1, 16)_ = 0.54, *p* = 0.47, Table 1].

### Osmometry and energetics

**Figure 1:**
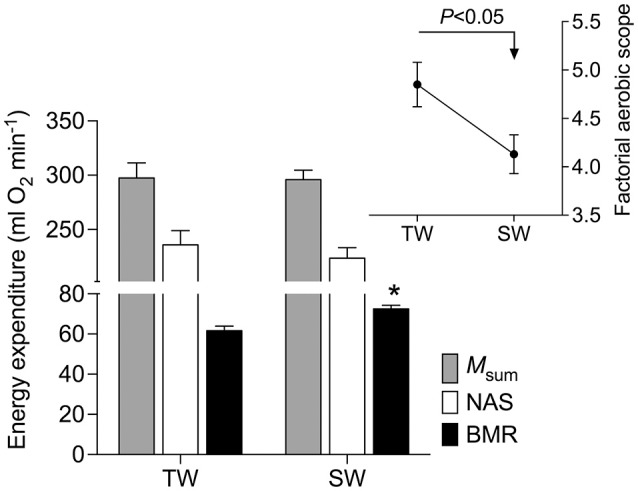
Rates of energy exenditure of *Zonotrichia capensis* after being acclimated to tap (TW) or 200 mM NaCl (SW) water for 30 days. An asterisk denotes significant differences after *a poseriori* Tukey test between treatments for each metabolic measurement. See text for abbreviations.

At the end of the acclimation period, plasma osmolality was similar between treatments [*F*_(1, 16)_ = 0.06, *p* = 0.81; Table 1], whereas the liquid phase of excreta was ~27% more concentrated in SW- vs. TW-acclimated birds [*F*_(1, 16)_ = 6.01, *p* = 0.027, Table 1]. As expected, BMR and M_sum_ were positively and significantly associated with body mass (*r*^2^ = 0.31, *p* = 0.016; *r*^2^ = 0.22, *p* = 0.049, respectively). BMR was 18% higher [*F*_(3, 16_ = 9.45, *p* = 0.0014, Figure 1] in SW- vs. TW-acclimated birds. M_sum_ did not differ significantly between SW- and TW-acclimated groups [*F*_(3, 16)_ = 1.77, *p* = 0.20, Figure 1], but FAS was significantly lower in SW- vs. TW-acclimated birds [*F*_(1, 17)_ = 6.09, *p* = 0.025, Figure 1]. Although NAS was ca. 6% lower in SW- vs. TW-acclimated birds, this difference was non-significant [*F*_(1, 17)_ = 3.11, *p* = 0.09, Figure 1]. FAS and NAS showed a strong significant linear relationship (*r*^2^ = 0.76, *p* < 0.0001). The time to hypothermia was not affected significantly by body mass (*r*^2^ = 0.09, *p* = 0.24) and did not differ significantly between SW- and TW-acclimated groups [*F*_(1, 16)_ = 0.95, *p* = 0.34, Table 1].

### Metabolic enzyme activity

**Figure 2:**
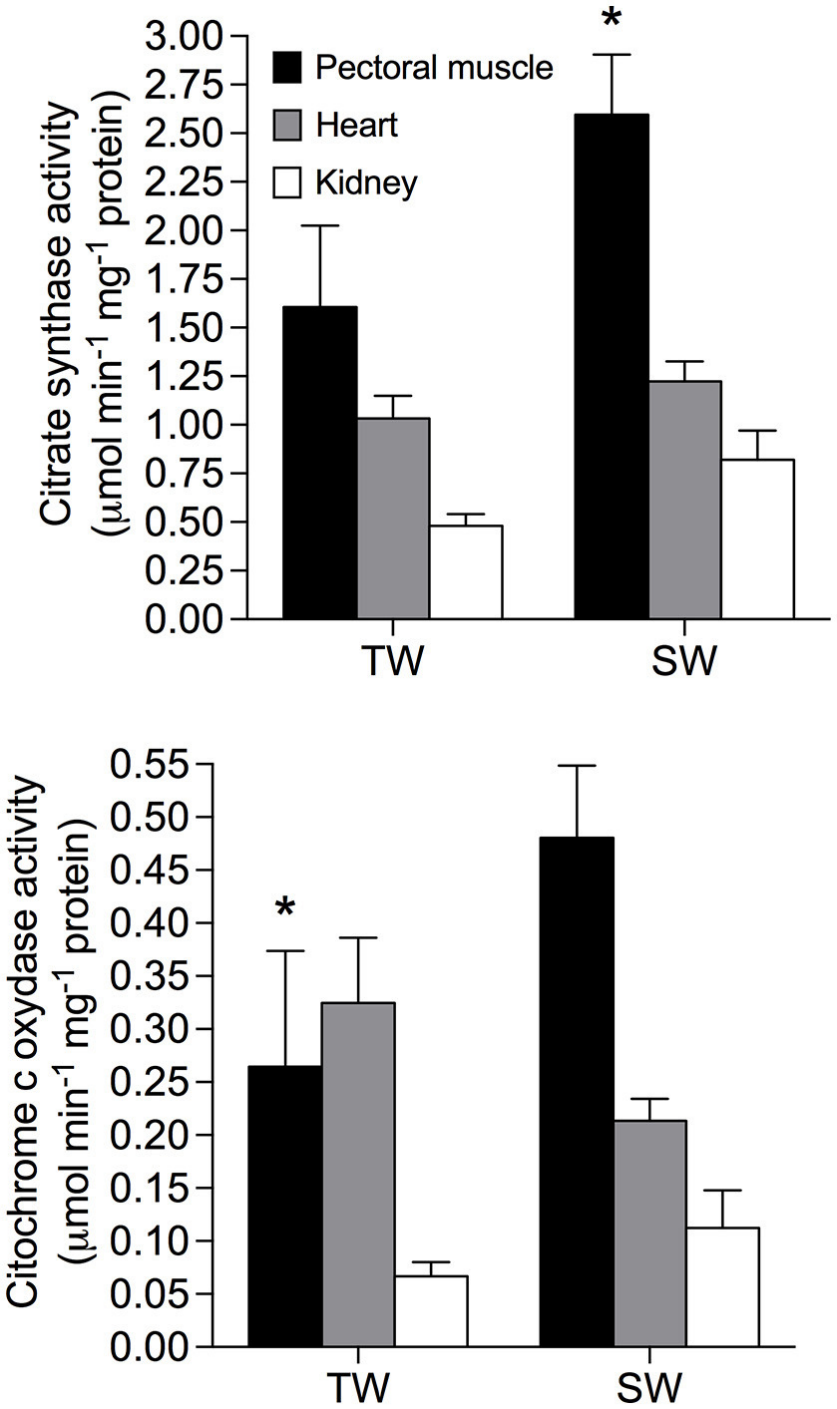
Metabolic enzymes in tissues of *Zonotrichia capensis* after being acclimated to tap (TW) or 200 mM NaCl (SW) water for 30 days. An asterisk denotes significant differences after *a poseriori* Tukey test between treatments for each tissue.

Repeated measures analyses revealed that metabolic enzyme activities varied among tissues and was influenced by drinking water treatment, but this effect depended on the enzyme and tissue analyzed (Figure 2). Citrate synthase activity was higher in SW- vs. TW-acclimated birds [*F*_(1, 15)_ = 6.49; *p* = 0.02], and was higher in pectoralis muscle than in heart and kidneys; [*F*_(2, 30)_ = 19.41; *p* < 0.001]. There was no significant interaction between tissue × treatment [*F*_(2, 30)_ = 1.60; *p* = 0.22]. *A posteriori* analyses revealed that difference between treatments was primarily explained by elevated enzyme activity in pectoralis muscle from birds in the SW-acclimated treatment. Cytochrome c-oxidase was unaffected by treatment [*F*_(1, 15)_ = 1.04; *p* = 0.32] but was significantly lower in kidney [*F*_(1, 15)_ = 12.68; *p* = 0.0001]. COX activity was influenced by the interaction between tissue and treatment [*F*_(2, 30)_ = 4.14; *p* = 0.02], and *a posteriori* analysis revealed that COX activity in pectoralis muscle explained the observed increase in the SW-acclimated treatment, (Figure 2).

### Oxidative parameters

**Figure 3:**
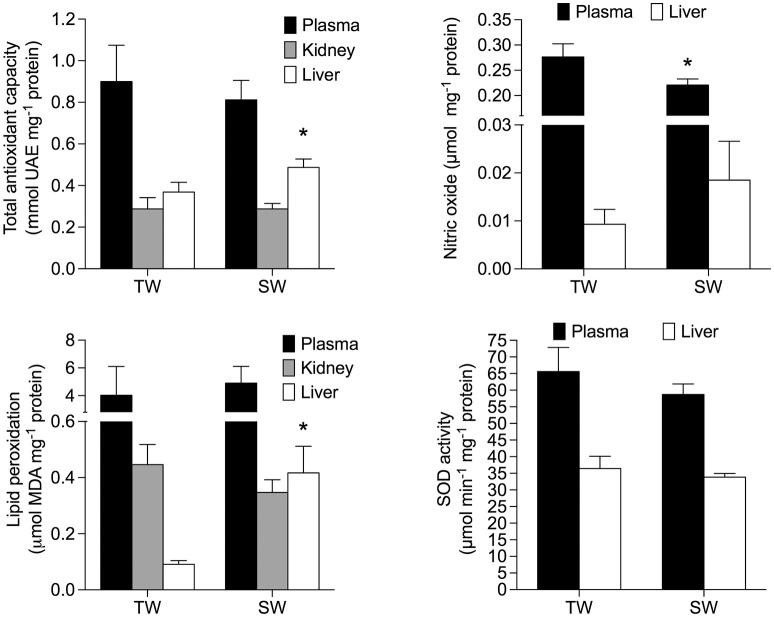
Oxidative stress parameters of *Zonotrichia capensis* after being acclimated to tap (TW) or 200 mM NaCl (SW) water for 30 days. Asterisks denote significant differences after *a poseriori* Tukey test between treatments for each tissue.

**Figure 4:**
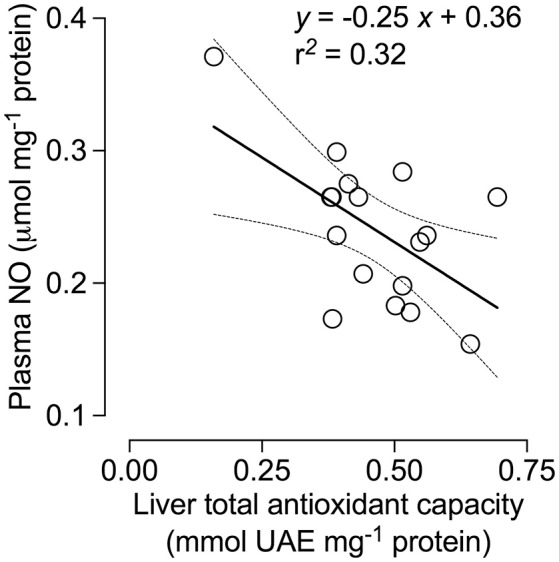
Nitric oxide (NO) concentration in the plasma of *Zonotrichia capensis* as a function of total antioxidant capacity (TAC) in the liver.

TAC activity was unaffected by treatment [*F*_(1, 14)_ = 0.97; *p* = 0.34] but was significantly different among all tissues [*F*_(2, 28)_ = 50.45; *p* < 0.0001]. TAC activity was also significantly affected by the interaction between treatment and tissues [*F*_(2, 28)_ = 5.96; *p* < 0.01], and was ~120% higher in the liver of SW- vs. TW-acclimated birds (Figure 3). We found no effect of drinking water treatment on [MDA] [*F*_(1, 15)_ = 0.25; *p* = 0.62], but [MDA] did significantly differ among tissues [*F*_(2, 30)_ = 13.59; *p* < 0.001] and there was a significant interaction between tissue × treatment on [MDA] [*F*_(2, 30)_ = 6.37; *p* < 0.02; Figure 3]. *A posteriori* analysis revealed that lipid peroxidation in liver was more than 400% higher in SW- vs. TW-acclimated birds. Kidney [MDA] was not different between drinking water treatments (Figure 3). Nitric oxide concentrations were higher in TW- vs. SW-acclimated birds; however, this difference was only marginally significant [*F*_(1, 15)_ = 3.88; *p* < 0.06] [NO] was higher in plasma than liver [*F*_(1, 15)_ = 231.90; *p* < 0.0001]. For [NO], The interaction between tissue × treatment was marginally significant [*F*_(1, 15)_ = 4.41; *p* = 0.05], so we performed the *a posteriori* analyses that showed plasma [NO] was higher in TW- vs. SW-acclimated birds (Figure 3). Superoxide dismutase activity was not different among drinking water treatments [*F*_(1, 15)_ = 1.33; *p* = 0.27], nor was the interaction between tissue × treatment significant [*F*_(1, 15)_ = 0.46; *p* < 0.50], but was 80% higher in plasma than in liver [*F*_(1, 15)_ = 75.02; *p* < 0.0001, Figure 3]. Finally, we used multiple regression analyses between antioxidant capacities ([SOD], [TAC]) in plasma and liver as independent variables and [NO] in plasma as dependent variable, which showed that 28% of variation in plasma [NO] can be explained by liver [TAC] [*r*^2^ = 0.32 *F*_(1, 15)_ = 7.24 *p* = 0.016, Figure 4].

## Discussion

Our results confirm that *Z. capensis* tolerate the chronic consumption of moderate levels of salt; however, these conditions promote physiological changes across multiple scales from whole organism traits to biochemical capacities and oxidative status at the cellular level. Specifically, our results revealed that salt intake leads to increases in urine osmolality and BMR, and to a decrease in aerobic scope. This decrease in metabolic scope, however, was not accompanied by changes in M_sum_ nor in the aerobic performance in the cold. Changes in rates of energy expenditure were also coupled with an increase in the weight and activity of mitochondrial enzymes of some internal organs (e.g., kidney and heart) and tissues (e.g., pectoralis muscle and blood plasma). Lastly, increased salt intake elicited changes in oxidative status by increasing antioxidant capacity in some tissues and caused oxidative damage in others. All of these changes appear to be related to the elimination of excess electrolytes associated with increased salt intake.

### Differences in rates of energy expenditure: causes and consequences

Our results suggest that the significant increase in cost of maintenance (BMR) associated with increased salt intake significantly affected energy budget in *Z. capensis*, and this is likely related to the osmoregulatory costs associated with excretion of excess electrolytes (Peña-Villalobos et al., 2013). Specifically, increased BMR was positively correlated with of the size of internal organs, especially the kidney, and increased activity of metabolic enzymes in such organs. It has been reported that changes in the mass and enzymatic activity of metabolically-demanding organs in birds (e.g., pectoralis muscle, kidney, liver, heart, and gut) are linked to changes in BMR (see Vezina and Williams, 2005; Swanson, 2010; Peña-Villalobos et al., 2014, 2017). In regards to enzyme activity, CS ad COX activities were higher in tissues of SW-acclimated than TP-acclimated birds (Figure 2). Overall, these patterns suggest that the observed increases in BMR was driven by tissue-specific metabolic demands.

We expected that both M_sum_ and aerobic scope would be influenced by the increased cost of osmoregulation associated with higher salt intake. Similarity in M_sum_ among treatments suggests there are no trade-offs between osmoregulatory and thermoregulatory abilities. This proposition is further supported by the absence of differences in the aerobic performance and cold tolerance between experimental groups. In contrast, we found a significant decrease in the aerobic scope (FAS) of SW-acclimated birds, which is likely caused by increases in BMR given that M_sum_ was similar between treatments. FAS is a robust trait for characterizing animal performance (Killen et al., 2016; Nespolo et al., 2017) because they estimate the energy available to drive a range of key functions, such as growth and reproduction (e.g., Guderley and Pörtner, 2010; Maldonado et al., 2016; Nespolo et al., 2017; Auer et al., in press). Thus, our experiment shows that the higher energetic costs of osmoregulation associated with increased salt intake induced a decrease in the aerobic scope of *Z. capensis*, but there is no evidence that FAS reduction has an impact on other animal energy demanding activities, such as shivering endurance.

Inherent to the hypothesis regarding the existence of a trade-off between osmoregulation and thermoregulation, there is an implicit assumption that energy intake is invariant. However, some studies in birds revealed that metabolic ceiling is a flexible trait (Klaassen et al., 2004; Sgueo et al., 2012; Careau et al., 2014), so that an increase in the maintenance costs produced by the greater osmoregulatory expenditure can be compensated by a greater energy intake—until a limit given by digestive constraints—without necessarily compromising other functions. Unfortunately we do not consider the daily energy intake in our experimental design, which deserves more attention in the future. For example, it would be interesting to evaluate the extent to which daily energy expenditure is influenced by the use of marine/salty resources in migratory birds or in populations inhabiting environments with different degrees of salinity (e.g., Gutiérrez et al., 2012).

There is a growing agreement that M_sum_ mainly depends on the metabolic intensity and the mass of skeletal (e.g., pectoralis and heart) muscles (Swanson, 2010; Zheng et al., 2013, but see Barceló et al., 2017). Thus, the finding that M_sum_ remain unchanged between experimental groups while enzyme activities increased 60–80% in pectoralis muscle of SW- vs. TW-acclimated birds seems to be contradictory. However, there is increasing evidence suggesting that drivers of M_sum_ flexibility are not uniform across species. For example Swanson et al. (2014) reported that pectoralis CS activities were significantly positively correlated with M_sum_ for *Passer domesticus*, but not for *Junco hyemalis*, whereas Sgueo et al. (2012) reported that the Northern Cardinal (*Cardinalis cardinalis*) modified its metabolic capacity significantly during winter, in comparison to summer acclimatized birds. However, CS in pectoralis remained unchanged. Moreover, avian metabolic capacities are not only affected by the mass and metabolic intensity of skeletal muscles, but also by several limiting processes, such as lung volume, cardiovascular efficiency, blood oxygen carrying capacity, among others (see Swanson, 2010 for a review). Thus, it is possible that an increase in metabolic enzymes concentration is a potential, but not required, mechanism to elicit changes in M_sum_.

### Oxidative status

One of the objectives of our study was to assess whether our model organism experienced increased oxidative stress or altered antioxidant enzyme activities because increased of salt intake. Oxidative status is an important fitness-related trait because of its role in controlling reproductive performance and mortality (Costantini and Møller, 2009; Costantini, 2014). Our study revealed that *Z. capensis* acclimated to salt water undergo adjustments in oxidative status, as shown by changes in TAC and lipid peroxidation (Figure 3). To protect against oxidative damage or stress, organisms usually repair or remove ROS-damaged molecules by creating endogenous antioxidant barriers (e.g., SOD and catalase) and exogenous agents (uric acid) (Costantini, 2014); thus, oxidative stress occurs when ROS production exceeds antioxidant capacity (Harman, 1956). Our results show that TAC activity increased by ~40% in the liver of SW-acclimated relative to TW-acclimated birds (Figure 3). This pattern was not paralleled by changes in SOD activity, which suggests that TAC activity is driven by other endogenous and/or exogenous sources of antioxidant capacity.

Our results also revealed that SW-acclimated *Z. capensis* showed clear signs of oxidative damage as evidenced by lipid peroxidation in the liver. Coupled with the observed changes in TAC in the liver, this pattern agrees with previous studies that suggest the liver is sensitive to exercise-induced oxidative stress, and can quickly adapt to such conditions via antioxidant responses (Radak et al., 1999, 2001). Our results also show that not all tissues responded in the same way in regards to antioxidant response and oxidative damage, which has been previously reported in other taxa (e.g., Costantini and Møller, 2009; Costantini et al., 2010; Beaulieu and Costantini, 2014), and confirms that the response of animals to environmental stressors is complex. One example of such complexity is the counterintuitive patterns we observed in NO concentrations, a free radical that was present in all tissues we evaluated. NO concentration was slightly but not significantly higher in the liver of SW-acclimated vs. TW-acclimated birds (Figure 3). In contrast, NO concentration was significantly higher in plasma of TW-acclimated vs. SW-acclimated animals. The negative correlation we found between TAC in liver and NO concentration in plasma suggests that consistent ROS production resulting from salt acclimation efficiently up-regulates the liver repair system. These conditions may have led to a decrease in ROS levels that are below those observed in previous salt acclimatization experiments. This kind of overcompensation (see Radak et al., 2001) has been reported in nematodes, in which the antioxidant systems may overcompensate for increased rates of ROS production that ultimately results in lower oxidative damage to tissues (Brys et al., 2007).

### Metabolism and oxidative stress

Several studies have reported that a change in abiotic environmental conditions (e.g., ambient temperature) produces significant increases in an animal's metabolic rate that in turn may modify its oxidative status via increases in the production of ROS and/or antioxidant defenses (Commoner et al., 1954; Ji, 1999; Tumminello and Fuller-Espie, 2013; Casasole et al., 2017). For instance, the exposure of two passerines (*Passer domesticus* and *Dumetella caroliniensis*) to low environmental temperatures produced an increase in antioxidant capacity (Cohen et al., 2008), whereas increased resting metabolic rate and levels of oxidative protein damage were observed in voles (*Microtus agrestis*) exposed to cold stress (Selman et al., 2002). We found SW-acclimated *Z. capensis* had higher metabolic rates resulting from higher osmoregulatory demands. Such increases in metabolic rates could lead to an overproduction of ROS. This is also evidenced by the overall increase in the mitochondrial enzyme activities, namely CS and COX, in almost all of the tissues we evaluated (Figure 2). Mitochondria are the principal source of cellular ROS associated with the production of superoxide anions as byproducts of the electron transport chain and other redox reactions (Srinivasan and Avadhani, 2012). Thus, the activity of mitochondria enzymes, such as CS and COX seems to be a likely mechanism responsible for changes in the oxidative status we observed in SW-acclimated *Z. capensis*.

## Conclusions

When acclimated to drinking water of moderate salinity (200 mM NaCl), *Z. capensis* exhibited several physiological responses, including (1) adjustments in osmoregulatory function via increased urine osmolality, (2) modifications in energy budget as evidenced by increased BMR and decreased aerobic scope, (3) changes of cellular metabolism as shown by increases in metabolic enzyme activities, and (4) alterations in the oxidative status, namely increases in antioxidant defenses and lipid peroxidation. We propose that the physiological changes observed in *Z. capensis* acclimated to saltwater could be a common phenomena in birds, and likely explains selection of prey containing little salt and habitats associated with low salinity, even in species (gulls and pelicans) with functional salt glands (Mahoney and Jhel, 1985; Nyström and Pehrsson, 1988; Hughes and Winkler, 1990; Rubega and Robinsons, 1997; Gutiérrez, 2014; Troup and Dutka, 2014) that avoid the physiological consequences of salt intake. It has also been suggested that oxidative stress could have a significant impact on some components of fitness in wild animals (Costantini, 2014), so that modifications in the oxidative status by salt consumption could affect the reproductive potential and survival of free-ranging birds. An interesting question that warrants further exploration is whether there are differences in the responsiveness of the antioxidant capacity of bird species (Rainio et al., 2013) that use different proportions of saline (marine) resources.

## Author contributions

Conception and design: PS, JS, SN, RN, and FB. Data acquisition and analysis: PS, CC, CN, IP, and KM. Data interpretation: FB, KM, PS, RN, and SN. All authors collaborated with the draft of the work and approved the final version of the manuscript.

### Conflict of interest statement

The authors declare that the research was conducted in the absence of any commercial or financial relationships that could be construed as a potential conflict of interest.
